# Prognostic Value of Histone Modifying Enzyme EZH2 in RCHOP-Treated Diffuse Large B-Cell Lymphoma and High Grade B-Cell Lymphoma

**DOI:** 10.3390/jpm11121384

**Published:** 2021-12-18

**Authors:** Sara Petronilho, José Pedro Sequeira, Sofia Paulino, Paula Lopes, Susana Lisboa, Sérgio Chacim, João Lobo, Manuel Teixeira, Carmen Jerónimo, Rui Henrique

**Affiliations:** 1 Department of Pathology, Portuguese Oncology Institute of Porto (IPO Porto), R. Dr. António Bernardino de Almeida, 4200072 Porto, Portugal; sara.petronilho@ipoporto.min-saude.pt (S.P.); sofia.paulino@ipoporto.min-saude.pt (S.P.); paulalopes@ipoporto.min-saude.pt (P.L.); jpedro.lobo@ipoporto.min-saude.pt (J.L.); 2 Cancer Biology and Epigenetics Group, Research Center of IPO Porto (CI-IPOP)/RISE@CI-IPOP (Health Research Network), Portuguese Oncology Institute of Porto (IPO Porto)/Porto Comprehensive Cancer Center (Porto.CCC), R. Dr. António Bernardino de Almeida, 4200-072, Porto, Portugal; jose.leite.sequeira@ipoporto.min-saude.pt (J.P.S.); sergio.chacim@ipoporto.min-saude.pt (S.C.); 3 Department of Genetics, Portuguese Oncology Institute of Porto (IPO Porto), R. Dr. António Bernardino de Almeida, 4200072 Porto, Portugal; susanalisboa@ipoporto.min-saude.pt (S.L.); manuelteixeira@ipoporto.min-saude.pt (M.T.); 4 Department of Hematology, Portuguese Oncology Institute of Porto (IPO Porto), R. Dr. António Bernardino de Almeida, 4200072 Porto, Portugal; 5 Department of Pathology and Molecular Immunology, Institute of Biomedical Sciences Abel Salazar, University of Porto (ICBAS-UP), Rua Jorge Viterbo Ferreira 228, 4050-513 Porto, Portugal

**Keywords:** DLBCL, HGBCL, RCHOP, epigenetics, histones, prognostic, EZH2

## Abstract

Background: DLBCL represent a heterogeneous group of aggressive diseases. High grade B-cell lymphomas (HGBCL) were recently individualized from DLBCL as a discrete diagnostic entity due to their worse prognosis. Currently, although most patients are successfully treated with RCHOP regimens, 1/3 will either not respond or ultimately relapse. Alterations in histone modifying enzymes have emerged as the most common alterations in DLBCL, but their role as prognostic biomarkers is controversial. We aimed to ascertain the prognostic value of EZH2 immunoexpression in RCHOP-treated DLBCL and HGBCL. Results: We performed a retrospective cohort study including 125 patients with RCHOP-treated DLBCL or HGBCL. EZH2 expression levels did not differ between diagnostic groups or between DLBCL-NOS molecular groups. We found no associations between EZH2 expression levels and outcome, including in the subgroup analysis (GC versus non-GC). Nonetheless, EZH2/BCL2 co-expression was significantly associated with worse outcome (event free survival and overall survival). Conclusion: Although EZH2 mutations are almost exclusively found in GC-DLBCL, we found similar EZH2 expression levels in both DLBCL-NOS molecular groups, suggesting non-mutational mechanisms of EZH2 deregulation. These findings suggest that the use of EZH2 antagonists might be extended to non-GC DLBCL patients with clinical benefit. EZH2/BCL2 co-expression was associated with a worse outcome.

## 1. Introduction

Diffuse large B-cell lymphomas (DLBCL) constitute a heterogeneous group of aggressive B-cell lymphomas that are clinically, pathologically and genetically diverse [1]. Treatment with the current standard immunochemotherapy regimen comprising rituximab, cyclophosphamide, hydroxydaunorubicin, vincristine and prednisone (RCHOP) is curative in up to 70% of patients, but almost one third remain refractory to initial therapy or ultimately relapse [2].

Owing to the disease’s heterogeneous clinical course, the ability to predict outcome is vital. Although several clinical, radiologic, pathologic and cytogenetic features have been pointed out as potential prognostic biomarkers in DLBCL, studies have so far produced inconsistent findings, and validation as independent parameters is still mostly lacking [1,2]. Possible explanations for these often-conflicting results reflect both design issues, including heterogeneity of the population and treatment modalities, and technical/interpretative factors, namely, the use of different evaluation methods and reference values. The new diagnostic category of high grade B-cell lymphomas (HGBCL) emerged from this pursuit of relevant prognostic biomarkers. It is considered as a distinct entity, which for biological and clinical reasons must be separated from DLBCL-NOS. HGBCL comprise two different groups of patients: HGBCL-NOS (encompassing cases that either have features intermediate between DLBCL and Burkitt Lymphoma or appear blastoid) and “double-hit/triple hit” HGBCL (HGBCL-DH/TH), which includes cases presenting with MYC and BCL2 and/or BCL6 rearrangements [1].

In recent years, with the emergence of next generation sequencing (NGS) based studies, many alterations in DLBCL have been described, some withholding promising value as prognostic and/or predictive biomarkers [3]. Among the most commonly mutated genes are those coding for histone modifying enzymes, which collectively emerge as the most frequent alterations in DLBCL, present in over one third of cases, independently of disease subtype [3]. EZH2 is a HMT (histone methyltransferase) which specifically tri-methylates lysine-27 of histone 3 (H3K27me3). EZH2 mutations arise almost exclusively in Germinal Center (GC) subtype DLBCL, being present in 22% of GC-DLBCL [4], and represent gain-of-function mutations which occur mostly at hotspot Y641 [5,6,7]. Thus, allele-specific EZH2 inhibitors are being exploited as therapeutic strategies in EZH2 mutated DLBCL patients. In fact, several phase 1 and 2 clinical trials testing EZH inhibitors in relapsed/refractory GC DLBCL are currently in progress [2,8]. Interestingly, some studies have shown that high-level expression of EZH2 and accumulation of H3K27me3 occur even in the absence of EZH2 mutation, raising the possibility of non-mutational mechanisms causing EZH2 deregulation [9,10].

**Figure A1:**
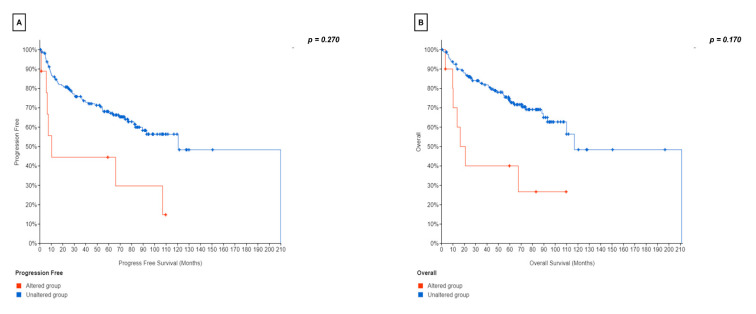
Univariate analysis of Progression Free Survival (**A**) and Overall Survival (**B**) regarding EZH2 status. EZH2 mutated DLBCL is associated with worse Overall and Progression Free Survival, albeit not significantly. Data and graphs were generated from the platform cBioPortal [4] and comprise 237 patients included in two studies (DFCI, Nat Med 2018; TCGA, PanCancer Atlas) with publicly available information regarding EZH2 status and follow-up.

Despite the well-established mutational status and driver function of this gene in DLBCL, the prognostic value of its mutational status remains controversial, with the few published data mostly pointing towards a lack thereof [7,11]. In spite of this, analysis of publicly available data from patients included in two different studies [5]—warranting cautious interpretation, given the potential use of different inclusion and outcome criteria-associates EZH2 mutated DLBCL with worse Overall and Progression Free Survival, albeit not significantly (Figure A1) Furthermore, when considering EZH2 expression, some studies suggest that it may be a promising biomarker in DLBCL [12,13,14,15,16,17,18,19]. Nonetheless, published data are inconsistent, possibly due to the use of different methodologies (universally semiquantitative), different positivity threshold, inclusion of heterogeneously treated patients and small number of patients per study (median number of patients per study: 68, interquartile range: 37 to 92). Regarding HGBCL in particular, to the best of our knowledge, the prognostic value of EZH2 mutational status or EZH2 expression has not been reported.

Herein, we aimed to ascertain the prognostic value of EZH2 immunoexpression in a large RCHOP-treated DLBCL/HGBCL patient cohort, using a digital imaging system for quantification.

## 2. Materials and Methods

### 2.1. Study Population, Inclusion and Exclusion Criteria

**Figure A2:**
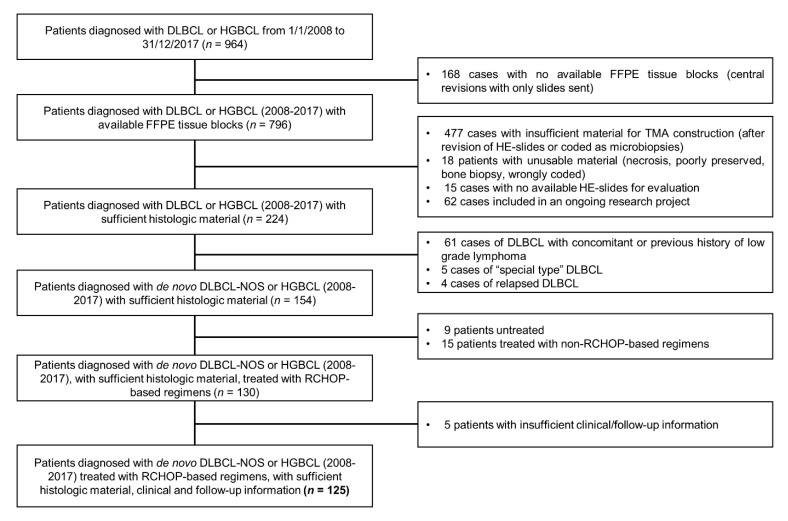
Flow chart of the patients included in the study.

The present study is a retrospective cohort study. As DLBCL/HGBCL are aggressive neoplasms, most refractory/relapsed disease occurs within the first two years after diagnosis [2]. We retrospectively identified patients with de novo DLBCL or HGBCL diagnosed at the Portuguese Oncology Institute of Porto (IPO Porto), Portugal, from 1 January 2008 to 31 December 2017, ensuring a minimum follow up period of 2 years by the time of statistical analysis. Our inclusion and exclusion criteria are summarized in Table A1. In all included cases for which material was collected in other hospitals, a formal request for the use of material in a research project was sent. After review of H&E-stained slides, tissue blocks and clinical charts, 125 patients were included in the final study cohort (Figure A2). The patients who fulfilled the inclusions criteria were mostly similar to the whole cohort regarding sex (42% vs. 45% males, respectively) and age distribution (median age of 66 in both groups). We observed some differences regarding the prevalence of HGBCL (9% vs. 2%) and the distribution of GC (40% vs. 49%) and non-GC (60% vs. 51%) subgroups within DLBCL-NOS. These differences are mainly explained by the absence of available data on MYC, BCL2 and BCL6 rearrangement status in most of the (non-included) cases, allied to the fact that HGBCL–DH/TH most frequently present a GC phenotype. Furthermore, there was no information regarding MUM1 status in some CD10-negative cases regarding the whole cohort, precluding classification of these (likely non-GC subtype) cases.

### 2.2. CASE Selection and EZH2 Immunohistochemistry

**Figure 1:**
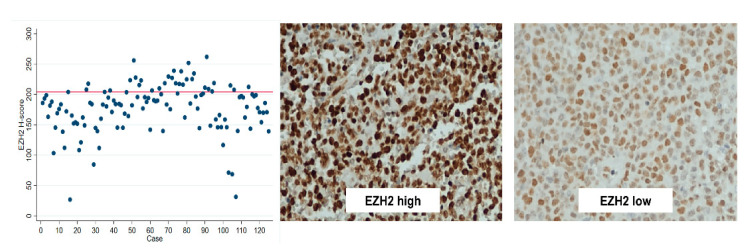
Distribution dotplot of EZH2 H-score (*n* = 125) and illustrative cases of high versus low expression levels. Immunostaining was performed in TMAs, and IHC nuclear staining quantification was performed using a digital system (GenASIS™, Israel). Evaluation of intensity (score 0–3) and % of positive cells (0–100) was performed, allowing the calculation of an H-Score (0–300). Expression level H-scores were dichotomized in high *versus* low expression using the 75th percentile (204) as cut-off point. The staining pattern for EZH2 was homogeneous within each case. In most cases, there was intermediate to strong staining in the majority of tumor cells. Thus, the use of a digital software proved invaluable to allow the detection of small differences, which may not have been apparent had the authors used an “eyeball” evaluation strategy.

Tissue microarrays (TMAs) were constructed after delimitation of the tumor area by a pathologist, each including 3 cores per case (23 cores per TMA, including two cores of liver parenchyma and one core of tonsil, as positive controls). EZH2 immunostaining was performed in the TMAs, after optimization at our laboratory. Briefly, after antigen retrieval for both antibodies with citrate buffer (pH 6.0) in microwave (20 min), endogenous peroxidase activity was blocked with 0.6% hydrogen peroxide and normal horse serum (dilution 1:50) was used to blocked non-specific reactions. TMAs were incubated overnight with antibody EZH2 (Product Code NCL-L-EZH2, dilution 1:200, Leica Biosystems, United Kingdom); post-primary antibody and polymer were incubated for 30 min at room temperature (Novolink™ Polymer Detection System—Novocastra, Product No. RE7150-K). Diaminobenzidine was the chromogen used and slides were counterstain with hematoxylin. As positive controls, we used normal testicular tissue. IHC nuclear staining evaluation was performed by the same pathology trainee, blinded to all clinicopathological information, using a digital imaging system (GenASIS™, Israel). Evaluation of a median of 2657 cells (interquartile range 2360 to 3220) per case for intensity (score 0–3) and % of positive cells (0-100) was performed, allowing the calculation of the H-Score (1 × (% cells 1+) + 2 × (% cells 2+) + 3 × (% cells 3+)). H-score results (0–300) were dichotomized into high versus low expression using the 75th percentile (EZH2 > 204) as cut-off point. (Figure 1). This represented the cut-off point which provided the best discrimination between low and high EZH2 expression groups relating to patient outcome.

### 2.3. Clinical, Morphological and Immunohistochemical Baseline Characteristics

All slides were reevaluated to assess for potential prognostically relevant histological features (immunoblastic, pleomorphic and high-grade morphology), to determine the molecular group (GC or non-GC subtype, based on the expression of CD10, BCL6 and MUM1 according to the Hans algorithm) [20], to ascertain CD5-positivity (10% threshold), and double expressor status—including both BCL2-positivity (threshold set as 50% of moderate to strong immunostaining) and MYC-positivity (40% threshold) [21]. Of note, all CD5-positive cases were cyclin-D1 negative. Methods regarding CD20, CD3, CD5, BCL2, CD10, BCL6, MUM1 and MYC immunostaining can be found in the Appendix A. Concordant/discordant bone marrow involvement status, clinical and radiological information were collected from the patients’ clinical charts and original pathology report.

### 2.4. Fluorescent In Situ Hybridization

Sample processing, hybridization and analysis were performed as previously described [22] using dual color, break apart probes flanking the BCL6 (3q27), MYC (8q24) and BCL2 (18q21) genes (Zytovision, Bremerhaven, Germany). Briefly, slides were deparaffinized, re-hydrated in 2× SSC, and placed in a solution of 1 mol/L sodium sulfocyanate at 80 °C for 10 min (Merck, Darmstadt, Germany). Following tissue digestion with 6 mg/mL pepsin (Sigma-Aldrich, Steinheim, Germany) for 6 min at 37 °C, slides were rinsed in 2× SSC and dehydrated in a series of ethanol. Ten µL of probe was applied to each slide and codenatured at 80 °C for 8 min. Hybridization took place for 18 h at 37 °C followed by post hybridization washes in 2× SSC/0.5% Igepal (Sigma-Aldrich) at 74 °C for 5 min and 2× SSC/0.1% Igepal at room temperature for 3 min. Slides were counterstained with DAPI and 100 morphologically intact, non-overlapping nuclei were scored.

### 2.5. Follow-Up and Endpoints

Follow-up data were retrieved from the patients’ clinical charts, including: date of histologically confirmed primary lymphoma diagnosis (defined as the date of the definitive pathology report issued in IPO Porto); date of response evaluation (defined as date of the first PET/CT showing complete imaging response); type of response (complete response or partial/no response); date of relapse (defined as the date of histologically confirmed lymphoma relapse or, when no biopsy was performed, date of PET/CT with evidence of disease); date of last follow-up; vital state at last follow-up (dead or alive, with or without evidence of disease); date and cause of death (with deaths occurring up to 3 months after complete response being considered as deaths due to disease).

The primary endpoint was event free survival (EFS), defined as time from diagnosis to relapse and/or death by disease. For patients with partial or no response to RCHOP-like regimens, EFS was considered as time from diagnosis to response evaluation and/or change of treatment. Our secondary endpoint was overall survival (OS), defined as time from diagnosis to death by any cause.

### 2.6. Statistical Analysis

We used the statistical software STATA 15^®^. Descriptive statistics were used for IHC description and the chi-square or t-test for subgroup analysis, as appropriate. We performed a global analysis including all patients (HGBCL-DH/TH; HGBCL–NOS; DLBCL–NOS), as well as a separate analysis encompassing only patients diagnosed with DLBCL-NOS. No separate analysis regarding HGBCL was performed, due to the low number of patients. Subgroup analyses per molecular group were performed in DLBCL-NOS (GC versus non-GC subgroups). Regarding time to event outcomes, a univariable analysis of each variable was performed. Given the recent results [23] regarding the prognostic value of the co-expression of EZH2 and BCL2 in DLBCL, we post hoc included this analysis in our work. We performed our multivariable model using a Cox proportional hazards analysis, including all clinical and pathological features demonstrating statistically significant associations with outcome in the univariate analyses. Significance was set at an alpha level of 0.05.

## 3. Results

**Figure 2:**
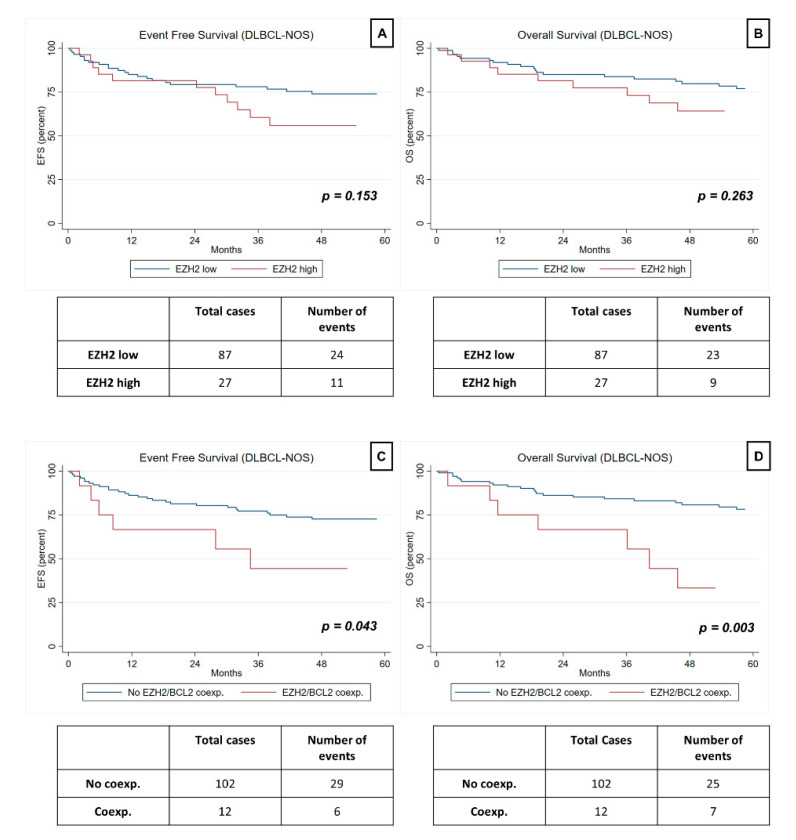
Univariate analysis of Event Free Survival (EFS) and Overall Survival (OS) in DLBCL-NOS. The displayed Kaplan Meier curves show comparison of EFS and OS with regard to: (**A**) and (**B**)-EZH2 expression levels; (**C**) and (**D**)-EZH2/BCL2 co-expression (coexp).

The clinical and pathological features of the 125 patients are summarized in Table 1. Results regarding our primary endpoint (EFS) and our secondary endpoint (OS) are summarized in Table 2 and Table 3, respectively, and graphically displayed in Figure 2.

### 3.1. Clinical Characteristics

Briefly, the cohort comprised 125 patients (Table 1), 58% of which were women. Median age was 66 years, ranging from 20 to 83 years. Most patients presented with advanced Ann Arbor stage (stages III or IV), and with low to intermediate-risk International Prognostic Index (R-IPI) score. Concordant bone marrow involvement was observed in 8/121 patients (7%). No significant differences in clinical parameters between patients with DLBCL-NOS GC and DLBCL-NOS non-GC were found.

### 3.2. Pathological and Cytogenetic Features

After morphological and cytogenetic analysis, 11/125 cases (9%) were established as HGBCL: 5 HGBCL-NOS and 6 HGBCL-DH/TH. The remaining 114 cases consisted of DLBCL-NOS, which were further classified based on the Hans algorithm: 40% were deemed of Germinal Center (GC) subtype and 60% of non-GC subtype.

BCL2-positivity was found in 56% of patients, whereas double-expressor lymphomas represented 17% of cases. When considering only DLBCL-NOS, BCL2-positive lymphomas and double-expressors were more frequent in the non-GC subtype (68% vs. 33%; *p* < 0.001; and 2% vs. 21%; *p* = 0.005). Of note, 5 out of the 6 HGBCL-DH/TH were also double-expressors. BCL2-positivity was significantly associated with BCL2 translocation (*p* = 0.007), with 14/16 (87.5%) BCL2-translocated cases being BCL2-positivity.

Median EZH2 H-score was 177.9, ranging from 26.7 to 261.9. No differences in EZH2 immunoexpression were observed between DLBCL-NOS and HGBCL, nor between molecular groups in DLBCL-NOS. Both BCL2 immunoexpression and EZH2/BCL2 co-expression were more frequent in HGBCL–DH/TH when compared to DLBCL-NOS (100% vs. 54%, *p* = 0.025 and 50% vs. 10%, *p* = 0.004, respectively).

All clinicopathological characteristics showed similar distribution in the dichotomous high/low EZH2 expression groups (Table A2).

### 3.3. Outcomes and Follow-Up

**Figure A3:**
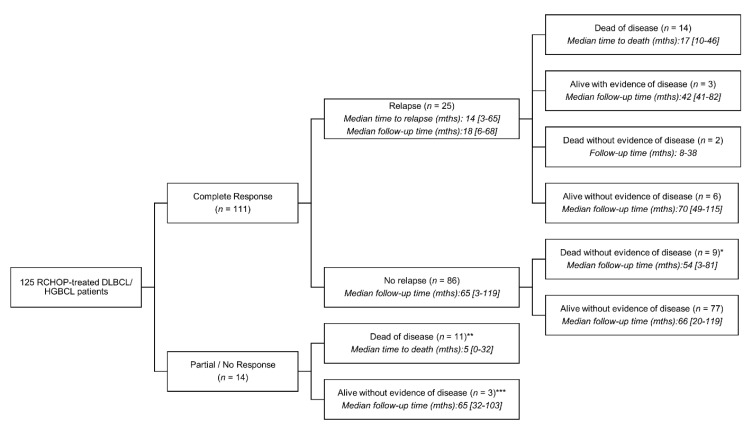
Response, outcome and follow up diagram of the 125 RCHOP-treated DLBCL/HGBCL patients included in the study. * includes 1 patient who died within the first 3 months after treatment (considered as dead of disease for statistical analysis purposes); ** includes 2 patients who underwent treatment changes at months 2 and 3 after diagnosis; *** all patients underwent treatment changes at months 1, 2 and 3 after diagnosis. RCHOP, Rituximab, cyclophosphamide, doxorubicin, vincristine, prednisone DLBCL, Diffuse large B-cell lymphoma; HGBC, High grade B-cell lymphoma; mths, months.

Outcome and follow-up data are summarized in Figure A3. Briefly, median follow-up time was 49 months (ranging from 0 to 119 months), and 40 events were noted regarding the primary endpoint. Five events occurred in patients with HGBCL and 35 in patients with DLBCL-NOS (6 in the GC and 29 in the non-GC subgroup). Of the 111/125 (89%) patients who achieved complete responses, 25 (23%) relapsed, 14/25 (56%) of which died of disease and 3/25 (12%) being alive with evidence of disease at time of manuscript preparation. Of the 14/125 patients (11%) who did not respond to first line RCHOP treatment, 11/14 (79%) died of disease. EZH2 expression was not significantly associated with treatment response (*p* = 0.787), with high EZH2 expression being present in 4/14 (29%) non-responders/partial responders and 28/111 (25%) complete responders.

### 3.4. Prognostic Value of EZH2 Expression in HGBCL and DLBCL-NOS

EZH2 expression was not significantly associated with EFS or OS in the whole cohort (HR 1.59, *p* = 0.173 and HR 1.57; *p* = 0.215, respectively), nor in DLBCL-NOS (HR 1.69, *p* = 0.153 and HR 1.56, *p* = 0.882, respectively). Regarding the subgroup analysis, high EZH2 expression was not significantly associated with EFS or OS in the non-GC subgroup (HR 2.11; 95%CI (0.97–4.57); *p* = 0.059 and HR 1.79; 95%CI (0.77–4.13); *p* = 0.174, respectively), nor in the GC subgroup (HR 0.71; 95%CI (0.08–6.15); *p* = 0.754 and HR 1.14; 95%CI (0.12–11.16; *p* = 0.909), respectively) (Table 2 and Table 3).

The limited number of HGBCL included in the study precluded a separate analysis of the prognostic value of EZH2 in this group of patients.

### 3.5. Prognostic Value of EZH2/BCL2 Coexpression in HGBCL and DLBCL-NOS

EZH2/BCL2 coexpression was independently associated with worse EFS in DLBCL-NOS (HR 3.43, *p* = 0.026), even after adjustment for BCL2-positivity and double-expressor status.

In univariable analysis, EZH2/BCL2 co-expression was significantly associated with worse EFS in the whole cohort (HR 2.40; *p* = 0.029) and in the DLBCL-NOS group (HR 2.50; *p* = 0.043), as well as with worse OS (HR 3.53; *p* = 0.001) in all patients. However, after adjustment for stage, R-IPI, bone marrow involvement, Hans molecular subtype, BCL2-positivity, double-expressor status and HGBCL-DH/TH diagnosis, these results did not reach significance.

## 4. Discussion

In the present study, we aimed to ascertain the prognostic value of EZH2 immunoexpression in de novo RCHOP-treated DLBCL-NOS and HGBCL patients. We did not find statistically significant associations between isolated EZH2 expression and outcome. However, we found an association between EZH2/BCL2 co-expression and worse outcome in these patients.

Regarding baseline clinicopathological data, our cohort is mostly comparable with previously published series [1]. Briefly, median age at diagnosis was 66 years, with just over half the patients presenting stage III or IV disease. Distribution by molecular group, BCL2-positivity, HGBCL–DH/TH and double-expressor rates were similar to those previously reported [3,21]. We did, however, observe some discordances, such as a minor female predominance (58%), a slightly lower incidence of bone marrow concordant involvement (6% in our series versus 10–25% expected value) and a higher incidence of CD5 positivity (14% versus 5–10% expected value). Regarding our primary outcome, 40/126 patients (32%) either did not respond to first line RCHOP treatment, relapsed or died from disease (median time to event: 9 months, interquartile range 4–28), which is within the expected range [2].

EZH2 is a HMT which specifically catalyzes mono, di and trimethylation of H3K27 [24]. Gain of function mutations of *EZH2* have been reported in several neoplasms, including DLBCL [3,5], leading to excessive H3K27me3, a repressive mark, and ultimately promoting decreased differentiation, dysregulated proliferation and lymphomagenesis [24]. Of note, high EZH2 expression levels and accumulation of H3K27me3 are common even in the absence of mutation [9,10,16,19], reflecting alternative mechanisms of *EZH2* deregulation. A possible explanation lies in *p*-ERK-related signaling and up-regulation of *EZH2* due to enhanced expression of BCL2 [18,20] (although our study did not show an association between EZH2 expression and BCL2 positivity; *p* = 0.428). The fact that we found similar levels of EZH2 expression in GC and non-GC DLBCL is thus not surprising, and follows previous publications [9,10,20].

Several reports [12,13,16,20] have described an association between EZH2 expression and patient outcome, mostly between high expression levels and worse prognosis [12,16,20]. As previously stated, we failed to find a statistically significant association between isolated EZH2 expression and patient outcome. Intriguingly, EZH2/BCL2 co-expression has recently been associated with worse EFS and OS [23]. Our data corroborate these findings, although further studies are needed to validate these results and clarify the underlying disease pathogenesis.

Adding to its putative prognostic value, EZH2 is also an actionable target, and several clinical trials evaluating the efficacy of anti-EZH2 drugs in refractory/relapsed DLBCL are currently ongoing [8]. Results of a phase 1 trial have shown promising antitumor activity of tazemetostat in patients with refractory B-cell non-Hodgkin lymphoma (both *EZH2* mutated and non-mutated) [25]. A subsequent phase 2 study confirmed the activity of tazemetostat in relapsed/refractory GC DLBCL, disclosing a higher response rate (29% vs. 15%) in patients with activating EZH2 mutations [26]. Following these results, tazemetostat is expected to obtain approval in relapsed or refractory DLBCL harboring activating *EZH2* mutations [27]. Nonetheless, as previously stated, we found similar and rather high EZH2 expression levels in both GC and non-GC DLBCL, which raises the question of whether treatment indication might be extended to non-mutated cases (of both molecular groups) in the future.

The present study was one of the largest conducted to ascertain the value of EZH2 expression as a biomarker in RCHOP treated DLBCL-NOS and HGBCL. The use of immunohistochemistry coupled with quantitative digital assessment as a primary evaluation method could be easily applied in routine practice. Indeed, it represents a rapid, inexpensive technique, and as the digital evaluation system starts to become widely available, it provides a more objective assessment when compared to the traditional “eyeball” evaluation. Of note, the staining pattern of EZH2 was very homogeneous, with most cases displaying intermediate to strong staining most of the tumor cells. Consequently, the use of a digital software proved invaluable, allowing the detection of small differences, which may have not been apparent had the authors used an “eyeball estimation” strategy.

Nevertheless, the general transversal limitations of biomarker validation studies still apply to the present work [28]. We conducted a retrospective study, tested multiple clinical endpoints and multiple biomarkers. These issues were addressed and minimized in the statistical analyses, notably by prioritizing a composite endpoint. Furthermore, the number of included patients and events was suboptimal, which limits the interpretation of some of the results, namely, regarding the multivariable analysis, and those regarding HGBCL and DLBCL-NOS subgroup analyses. Selection bias set by our inclusion criteria also likely occurred, as patients with aggressive disease (who died before start of treatment), and those who did not undergo standard of care due to severely deteriorated vital state were excluded, leading to potential underestimation of the number of events. However, these stringent criteria ensured the homogeneity of the cohort, which remains one of the most important study strengths.

## 5. Conclusions

We found similar expression levels of EZH in DLBCL-NOS and HGBCL, as well as in GC and non-GC DLBCL-NOS. These findings imply the existence of non-mutational mechanisms of EZH2 deregulation and suggest that EZH2 antagonists might be an option in the future treatment of these patients. Although we did not find a statistically significant association between isolated EZH2 expression and outcome, EZH2/BCL2 co-expression was associated with worse outcome in this cohort. Additional studies must be conducted to validate these results and further clarify the pathogenesis and prognostic implications of EZH2 dysregulation in DLBCL-NOS and HGBCL.

## Figures and Tables

**Table 1:** Baseline characteristics of the study cohort (*n* = 125).

	All Patients (*n* = 125)	DLBCL-NOS (*n* = 114)	DLBCL-NOS GC (*n* = 46)	DLBCL-NOS Non-GC (*n* = 68)	*p*
Male sex	52 (42%)	47 (41%)	23 (50%)	24 (35%)	0.118
Median age (years) <60 years	66 (20–83) 32 (26%)	66 (20–83) 29 (25%)	65 (23–80) 13 (28%)	66 (20–83) 16 (24%)	0.815 0.569
Stage III/IV	73 (58%)	66 (58%)	23 (50%)	43 (63%)	0.160
R-IPI 3–5 *	38 (31%)	35 (32%)	11 (24%)	24 (38%)	0.131
Bone marrow * Negative/Discordant Concordant	113 (93%) 8 (7%)	103 (94%) 7 (6%)	43 (93%) 3 (7%)	60 (94%) 4 (6%)	0.954
Morphology Centroblastic Immunoblastic Anaplastic High-grade	124 (91%) 1 (1%) 5 (4%) 5 (4%)	108 (95%) 1 (1%) 5 (4%) NA	45 (98%) 0 (0%) 1 (2%) NA	63 (93%) 1 (1%) 4 (6%) NA	NA
CD5-positive **	13 (13%)	10 (11%)	3 (8%)	7 (13%)	0.449
BCL2-positive	70 (56%)	61 (54%)	15 (33%)	46 (68%)	<0.001
MYC-positive ***	32 (27%)	25 (23%)	6 (14%)	19 (29%)	0.063
Double-expressor ***	20 (17%)	15 (14%)	1 (2%)	14 (21%)	0.005
HGBCL-DH/TH *MYC/BCL2* *MYC/BCL6* *MYC/BCL2/BCL6*	6 (5%) 3 (50%) 2 (33%) 1 (17%)	NA	NA	NA	NA
EZH2 Mean H-score EZH2 high	177.8 (26.7–261.9) 32 (26%)	176.8 (31.2–261.9) 27 (24%)	171.7 (68.5–256.0) 11 (24%)	180.2 (31.2–261.9) 16 (24%)	0.248 0.962
EZH2/BCL2 coexp.	16 (13%)	12 (11%)	2 (4%)	10 (15%)	0.077

* 4 missing values; ** 25 missing values; *** 5 missing values. *p* values compare DLBCL-NOS GC vs. nonGC.

**Table 2:** Univariate and multivariate * analysis of primary outcome (EFS) in DLBCL-NOS.

	All Patients *n* = 125				DLBCL-NOS *n* = 114			
	Univariate		Multivariate		Univariate		Multivariate	
	HR (95CI)	*p*	HR (95CI)	*p*	HR (95CI)	*p*	HR (95CI)	*p*
Male sex	0.89 (0.47–1.67)	0.711	*NA*	*NA*	1.20 (0.62–2.34)	0.584	*NA*	*NA*
Age (>60 years)	1.32 (0.63–2.78)	0.464	*NA*	*NA*	1.28 (0.58–2.82)	0.541	*NA*	*NA*
Stage (III/IV)	2.46 (1.20–5.03)	0.014	1.53 (0.60–3.90)	0.373	2.84 (1.29–6.26)	0.010	1.99 (0.73–5.40)	0.176
R-IPI (3–5)	2.07 (1.08–3.95)	0.028	1.12 (0.48–2.59)	0.799	2.11 (1.05–4.22)	0.036	1.03 (0.42–2.54)	0.942
BM concordant involvement	4.53 (1.87–11.00)	0.001	3.32 (1.14–9.69)	0.028	4.38 (1.66–11.51)	0.003	2.99 (0.91–9.88)	0.072
BCL2-positive	2.10 (1.07–4.14)	0.031	1.00 (0.44–2.25)	0.990	2.14 (1.05–4.38)	0.036	1.02 (0.44–2.34)	0.967
CD5-positive	0.96 (0.34–2.72)	0.933	*NA*	*NA*	0.93 (0.28–3.07)	0.905	*NA*	*NA*
GC subtype	0.38 (0.19–0.78)	0.009	0.44 (0.20–0.98)	0.046	0.26 (0.11–0.62)	0.003	0.31 (0.12–0.80)	0.016
HGBCL-DH/TH	2.39 (0.73–7.79)	0.148	2.25 (0.53–9.53)	0.272	*NA*	*NA*	*NA*	*NA*
HGBCL-NOS	1.77 (0.43–7.40)	0.431	*NA*	*NA*	*NA*	*NA*	*NA*	*NA*
Double- expressor	1.72 (0.82–3.62)	0.155	0.97 (0.37–2.58)	0.958	1.42 (0.59–3.42)	0.439	0.79 (0.25–2.47)	0.686
EZH2 high	1.59 (0.82–3.09)	0.173	*NA*	*NA*	1.69 (0.82–3.46)	0.153	*NA*	*NA*
*EZH2/BCL2* coexp.	2.40 (1.10–5.26)	0.029	1.93 (0.74–5.05)	0.178	2.50 (1.03–6.09)	0.043	2.01 (0.69–5.85)	0.200

* Multivariate analysis includes 115 (all cohort) and 105 (DLBCL-NOS) patients with complete information. In order to ascertain the independent prognostic value of EZH2/BCL2 coexpression, all variables significantly associated with outcome in the univariate analysis, as well as HGBCL-DH/TH and DE-status (given their association with BCL2 positivity and EZH2/BCL2 coexpression), were included in the model.

**Table 3:** Univariate and multivariate * analysis of secondary endpoint (overall survival).

	All Patients *n* = 125				DLBCL-NOS *n* = 114			
	Univariate		Multivariate		Univariate		Multivariate	
	HR (95CI)	*p*	HR (95CI)	*p*	HR (95CI)	*p*	HR (95CI)	*p*
Male sex	1.02 (0.52–1.98)	0.955	*NA*	*NA*	1.34 (0.67–2.68)	0.413	*NA*	*NA*
Age (>60 years)	3.58 (1.26–10.19)	0.017	4.58 (1.25–16.65)	0.021	4.33 (1.31–14.30)	0.016	7.33 (1.55–34.67)	0.012
Stage (III/IV)	3.92 (1.63–9.45)	0.002	3.54 (1.13–11.11)	0.030	4.30 (1.65–11.19)	0.003	4.94 (1.43–17.03)	0.011
R-IPI (3–5)	2.88 (1.45–5.74)	0.003	0.69 (0.31–2.04)	0.628	2.88 (1.38–6.01)	0.005	0.73 (0.27–1.98)	0.535
BM concordant involvement	4.42 (1.67–11.69)	0.003	1.94 (0.60–6.29)	0.269	4.26 (1.45–12.52)	0.008	1.40 (0.37–5.31)	0.625
BCL2-positive	2.72 (1.28–5.80)	0.009	1.22 (0.50–2.97)	0.654	2.47 (1.14–5.34)	0.021	1.18 (0.48–2.89)	0.722
CD5-positive	1.24 (0.43–3.59)	0.686	*NA*	*NA*	1.13 (0.34–3.77)	0.845	*NA*	*NA*
GC subtype	0.40 (0.19–0.85)	0.017	0.50 (0.21–1.16)	0.107	0.31 (0.13–0.75)	0.009	0.38 (0.15–0.99)	0.048
HGBCL–DH/TH	2.79 (0.85–9.18)	0.092	1.78 (0.41–7.66)	0.441	*NA*	*NA*	*NA*	*NA*
HGBCL-NOS	0.95 (0.13–6.95)	0.956	*NA*	*NA*	*NA*	*NA*	*NA*	*NA*
Double-expressor	1.70 (0.77–3.74)	0.188	1.00 (0.36–2.80)	0.999	1.26 (0.48–3.28)	0.636	0.65 (0.18–2.31)	0.503
EZH2 high	1.57 (0.77–3.23)	0.215	*NA*	*NA*	1.56 (0.72–3.40)	0.263	*NA*	*NA*
EZH2/BCL2 coexp.	3.53 (1.63–7.65)	0.001	2.70 (0.98–3.38)	0.054	3.70 (1.57–8.72)	0.003	3.43 (1.16–10.18)	0.026

* Multivariate analysis includes 115 (all cohort) and 105 (DLBCL-NOS) patients with complete information. In order to ascertain the independent prognostic value of EZH2/BCL2 coexpression, all variables significantly associated with outcome in the univariate analysis, as well as HGBCL-DH/TH and DE-status (given their association with BCL2 positivity and EZH2/BCL2 coexpression) were included in the model.

## Data Availability

The datasets used and/or analyzed during the current study are available from the corresponding author on reasonable request.

## Appendix A

**Table A1:** Outline of inclusion and exclusion criteria.

Inclusion Criteria	Exclusion Criteria
Patients over 18 years of age	DLBCL not included in the NOS category
First diagnosis of de novo DLBCL NOS or HGBCL	Relapsed DLBCL/HGBCL
Treatment with RCHOP based regimens	Previous history or concomitant diagnosis of other hematological malignancies, including low grade B-cell lymphoma
Available 2-year follow-up information	Previous treatment for hematological malignancies
Diagnosis, treatment and follow-up at IPO Porto	Treatment with non-RCHOP based regimens/ no treatment
	Unavailable slides or FFPE blocks
	Insufficient or poorly preserved histological material
	Bone marrow/bone biopsies.
	Insufficient clinical/follow-up information

DLBCL, Diffuse large B-cell lymphoma; NOS, Not otherwise specified; HGBCL, High grade B-cell lymphoma; RCHOP, Rituximab, cyclophosphamide, doxorubicin, vincristine, prednisone; FFPE, Formalin Fixed Paraffin Embedded.

**Table A2:** Baseline characteristics of the study cohort according to EZH2 expression.

	*EZH2* Low *(n = 93)*	*EZH2* High *(n = 32)*	*p*
Male sex	34 (37%)	18 (56%)	0.051
Median age (years) Age <60 years	66 25 (27%)	70 7 (22%)	0.576
Stage III/IV	56 (60%)	17 (53%)	0.483
R-IPI 3–5 *	27 (30%)	11 (35%)	0.570
Bone Marrow* None/Discordant Concordant	86 (96%) 4 (4%)	28 (87%) 4 (13%)	0.118
DLBCL-NOS GC-subtype	35 (40%)	11 (41%)	0.962
HGBCL-DH/TH	3 (3%)	3 (9%)	0.160
HGBCL-NOS	3 (3%)	2 (6%)	0.451
CD5-positive **	9 (12%)	4 (15%)	0.743
BCL2-positive	54 (58%)	16 (50%)	0.428
MYC-positive ***	21 (23%)	11 (39%)	0.085
Double-expressor ***	14 (15%)	6 (21%)	0.440

* 4 missing values; ** 25 missing values; *** 5 missing values.
